# Cognitive Ability as a Non-modifiable Risk Factor for Post-prostatectomy Urinary Incontinence: A Double-Blinded, Prospective, Single-Center Trial

**DOI:** 10.3389/fsurg.2021.812197

**Published:** 2022-01-10

**Authors:** Mathias Reichert, Ionel Valentin Popeneciu, Annemarie Uhlig, Lutz Trojan, Mirjam Naomi Mohr

**Affiliations:** Department of Urology, University Medical Center Göttingen, Göttingen, Germany

**Keywords:** prostate cancer, robot-assisted radical prostatectomy, predictor, post-prostatectomy incontinence, cognitive ability, nerve sparing

## Abstract

**Introduction:** Urinary incontinence (UI) is a wide-spread and feared side-effect of conventional or even robot-assisted laparoscopic prostatectomy (RALP) due to its high impact on patients' quality of life (QoL). Non-modifiable risk factors for UI have already been identified – on surgical and patient side. Yet, to our knowledge, focus thus far has not been placed on functional aspects regarding general cognitive ability.

**Materials and Methods:** This is an observational single-center, prospective, double-blinded evaluation of 109 RALPs performed between 07/2020 and 03/2021. All patients underwent a Mini Mental State Examination (MMSE) prior to surgery to evaluate their cognitive ability. Early post-prostatectomy incontinence (PPI) was evaluated using a standardized 1 h pad test performed 24 h after removal of the urinary catheter. The association between MMSE results and PPI were evaluated using univariate and multivariate logistic regression models.

**Results:** Multivariate logistic regression analyses identified MMSE results and nerve sparing (NS) as independent predictors for PPI in patients with an intermediate MMSE result (25–27 points) having a 3.17 times higher risk of PPI when compared to patients with a good MMSE result (≥28) (95% Confidence Interval (CI): 1.22–9.06, *p* = 0.023), while patients without NS had a 3.53 times higher risk of PPI when compared to patients with NS (95% CI: 1.54–11.09, *p* = 0.006).

**Conclusion:** A lower cognitive ability should be treated as a non-modifiable risk-factor for early PPI. In the future it could find its place as a clinical screening tool to identify patients who require more attention especially in the pre-, but also in the postoperative phase.

## Introduction

Radical prostatectomy, aside from percutaneous radiation or brachytherapy, still remains the gold standard for curative treatment of localized prostate cancer. Despite developments toward less invasive procedures, as seen in the progressive establishment of the robot-assisted laparoscopic prostatectomy (RALP), relevant side effects, such as urinary incontinence (UI) still occur. UI is one of the most pertinent unwanted effects after RALP as it has a substantial impact on patients' quality of life (QoL) and on their lifestyles overall (1).

Better visualization and performance of accurate incisions in a very narrowed surgical field as well as the enlarged view are some of the many propagated advantages of robot-assisted surgeries in comparison to open approaches. Regarding radical prostatectomy, the preservation of the external urethral sphincter can be performed more safely than when using a retropubic approach. Furthermore, the neurovascular supply of the membranous urethra can be spared more precisely—at least theoretically (2). However, a better outcome regarding UI in RALPs compared to open surgery is yet to be proven (3).

Some studies suggest predictors of post-prostatectomy incontinence (PPI) such as age at time of surgery, Body-Mass-Index (BMI), prostate volume, and a history of previous transurethral surgery on the prostate gland (4, 5).

Mechanisms leading to PPI are still not entirely understood. Nevertheless, age at time of surgery seems to be one of the most significant factors, since younger patients recover quicker and more completely (6). But it remains unclear whether physical fitness fully explains the observed effect on PPI. Besides the better physical fitness of younger men, which could contribute to the better prognosis, clinical observations lead to the assumption that mental health also has a positive influence on the postoperative continence outcome.

Ogurel et al. showed that there is a correlation between cognitive ability and the appearance of a disease and its course (7). They linked the Mini-Mental-State-Examination (MMSE) with diabetic retinopathy and demonstrated that a bad result in the MMSE correlates with a worse prognosis of the retinopathy. Flaker et al. demonstrated the same correlation between low MMSE results and an increase of the appearance of vascular events and bleeding in patients with atrial fibrillation under anticoagulation therapy (8).

In our clinical experience, after observing patients undergoing RALP, there appears to be a difference in the PPI occurrence between patients which cannot be explained by the common risk factors such as past surgical procedures, age, or body fitness. We suggest that there are further aspects aside from the already proven non-modifiable risk-factors, patient- and surgical-sited, which influence the outcome of UI after radical prostatectomy.

This study prospectively evaluates a possible correlation between cognitive ability and early PPI after RALP. To our knowledge this is the first study to examine this issue thus far.

## Materials and Methods

This is a single-institution, observational, double-blinded, and prospective evaluation of patients who underwent RALP in the department of urology at the University Medical Center Goettingen between 07/2020 and 03/2021. The institutional review board of the University Medical Center Goettingen approved this study.

The indication for RALP was localized prostate cancer detected by biopsy. All patients were staged according to current guidelines (German-S3-Guidelines, EAU-Guidelines, respectively) (9, 10).

Exclusion criteria were poor patient literacy, which could lead to incorrect, bad MMSE results, and an interdisciplinary board decision to initiate a multimodal therapy before surgery.

The outcome of interest was the occurrence of PPI and its possible predictors. Is cognitive ability a suitable predictor for PPI?

All patients were seen at least 1 day before surgery. After study inclusion they underwent the MMSE by Folstein et al. (11), always carried out by the same physician to prevent unequal distortion of the data. Surgeons and patients had no information about the test result (double-blinded). While 30 points are always the maximum a patient can achieve, the further MMSE subdivisions are defined inconsistently in literature. In this study, the cut-offs were defined based on clinical performance in the MMSE and the median (28+/-1.6), yielding the following subdivisions: 28–30 points = no cognitive deficit, 25–27 points = mild cognitive deficit, and ≤ 24 points = severe cognitive deficit.

During the preoperative assessment, patients were asked if they had performed pelvic floor training, instructed by physiotherapists, before surgery.

All RALPs were performed by one of three surgeons using the DaVinci SI system. All surgeons had an experience of at least more than 450 RALPs, each. The patient allocation was randomized. The surgical techniques, e.g., preservation and reconstruction of the pelvic floor, were standardized (e.g., Rocco Stitch, etc.) (12). Preservation of the neurovascular bundle was performed whenever the oncological option with respect to the guidelines and the intraoperative findings was given and the patient asked for it. Preoperative erectile function was evaluated by the standardized IIEF questionnaire. For oncological safety we performed a frozen section of the entire lateral part of the gland surfacing the neurovascular bundle (from urethra to the bladder neck) during the RALP. When there was a cancer-positive area of the margin, the corresponding bundle was resected. Nerve sparing (NS) was assigned categorially into “no NS,” “unilateral NS” and “bilateral NS.” A transurethral catheter was placed in all patients after surgery. No suprapubic catheters were used.

The post-operative course applied to the entire patient collective was standardized with regard to the use of analgesics, diet, and physiotherapy. The decision about the duration of the transurethral catheter placement was dictated by the surgeons on basis of the intraoperative course (planned for 5 or 7 days). Before removing the tube, a radiological control (cystogram/retrograde urethrocystography) was performed. If there was no leakage of the contrast medium at the vesicourethral anastomosis, the catheter was removed accompanied by standardized instructions by a physician to train the pelvic floor.

Patients stayed hospitalized for at least 24 h after catheter-removal and received standardized pelvic floor training by physiotherapists within 5 h after catheter removal. The patients documented their voiding conditions using a standardized micturition protocol for 24 h (voiding rates, amount per fraction, pad usage, etc.). The next day urinary incontinence/continence was examined again by a standardized 1 h pad test (13) (see Appendix 1). Before discharge, ultrasonography of post voiding residual urine volume (PVR) was performed.

MMSE results were also categorized into “good” (no cognitive deficit), “intermediate” (mild cognitive deficit) and “bad” (severe cognitive deficit).

Categorical variables were described with absolute number and corresponding percentage; continuous variables were described using mean with standard deviation (SD), and median with range. Statistical comparisons of categorical variables between groups were performed using the Chi square test. Continuous variables were compared using the Wilcoxon rank sum test or Fisher's exact test based on evaluation of normal distribution by the Shapiro Wilks test. Binary univariate and multivariable logistic regression was used for identification of predictors. Variables were considered for inclusion in multivariable models accordingly to their literature-based influence on the outcome and based on statistical significance (*p* < 0.1) from univariate logistic regression analysis and retained in the final multivariable model if *p* < 0.05. The final multivariable logistic regression models were assessed for goodness-of-fit (calibration) with the Hosmer-Lemeshow test (14), and for discrimination with the AUC statistic. All statistical analyses were performed with R version 3.6.3 (R Core Development Team, Vienna, Austria) and RStudio version 1.1.463 (RStudio Inc., Boston, MA). Statistical tests with *p* < 0.05 were considered significant. All p-values are two-sided.

## Results

109 patients, which were included into this study, were considered for statistical evaluation of their data since they had a complete postoperative follow up, meaning they showed a complete pad test and a sufficient micturition protocol.

The average loss of urine in the pad test ranged from 0 to 328 ml. We categorized the patient population in two groups: “Dry patients” with a loss of urine <2 ml (*n* = 45) vs. “wet patients” with a greater loss (*n* = 64).

Patients' characteristics of both groups are shown in Table 1, including their histopathological findings in the prostatectomy specimen.

**Table 1:** Patient characteristics between the “dry” and the “wet” group.

		**Total (*n* = 109)**	**Dry (*n* = 45)**	**Wet (*n* = 64)**	***p*-value**
Age (years)	Median (min; max)	65 (48;79)	64 (48;78)	66 (51;79)	0.11
BMI	BMI <24	11 (10.1%)	3 (27.3%)	8 (72.7%)	0.55
	BMI 24– <30	77 (70.6%)	35 (45.5%)	42 (54.5%)	
	BMI 30– <35	17 (15.6%)	6 (35.3%)	11 (64.7%)	
	BMI ≥35	4 (3.7%)	1 (25.0%)	3 (75.0%)	
iPSA (ng/ml)	<4	7 (6.4%)	2 (28.6%)	5 (71.4%)	0.05
	4– <10	67 (61.5%)	32 (47.8%)	35 (52.2%)	
	10– <20	22 (20.2%)	10 (45.5%)	12 (54.5%)	
	≥20	13 (11.9%)	1 (7.7%)	12 (92.3%)	
Prostate volume (ml)	<40	52 (47.7%)	21 (40.4%)	31 (59.6%)	0.66
	40–90	54(49.5%)	22(40.7%)	32 (59.3%)	
	>90	3 (2.8%)	2 (66.7%)	1 (33.3%)	
IPSS (preoperative)	<8	62 (58.5%)	31 (50.0%)	31 (50.0%)	0.11
	8–19	37 (34.9 %)	13 (35.1%)	24 (64.9%)	
	20–35	7 (6.6%)	1 (14.3%)	6 (85.7%)	
ICIQ (preoperative)	No incon.	76 (76.0%)	33 (43.4%)	43 (56.6%)	0.77
	Light incon.	17 (17.0%)	7 (41.2%)	10 (58.8%)	
	Mid incon.	2 (2.0%)	1 (50.0%)	1 (50.0%)	
	Severe incon.	5 (5.0%)	1 (20.0%)	4 (80.0%)	
pT status	pT2	17 (15.6%)	8 (47.1%)	9 (52.9%)	0.8
	pT3-4	92 (84.4%)	37 (40.2%)	55 (59.8%)	
pN status	pN0	100 (92.6%)	42 (42.0%)	58 (58.0%)	0.57
	pN1	8 (7.4%)	2 (25.0%)	6 (75.0%)	
R status	R0	79 (72.5%)	35 (44.3%)	44 (55.7%)	0.46
	R1	29 (26.6%)	10 (34.5%)	19 (65.5%)	
	R2	1 (0,9%)	0	1 (100%)	
GS	6	3 (2,8%)	1 (33.3%)	2 (66.7%)	0.50
	7	80 (73.4 %)	35 (43.8%)	45 (56.2%)	
	8	7 (6.4%)	1 (14.3%)	6 (85.7%)	
	9	19 (17.4%)	8 (42.1%)	11 (57.9%)	
NS	Unilateral NS	35 (32.1%)	21 (60.0%)	14 (40.0%)	0.01
	Bilateral NS	35 (32.1%)	15 (42.9%)	20 (57.1%)	
	No NS	39 (35.8%)	9 (23.1%)	30 (76.9%)	
Pelvic floor training preoperative	Yes	17 (15.6%)	9 (52.9%)	8 (47.1%)	0.54
	No	53 (48.6%)	20 (37.7%)	33 (62.3%)	
	Unknown	39 (35.8%)	16 (41.0%)	23 (59.0%)	
MMSE	Good	70 (66.7%)	34 (48.6%)	36 (51.4%)	0.01
	Intermediate	33 (31.4%)	7 (21.2%)	26 (78.8%)	
	Bad	2 (1.9%)	2 (100%)	0	
Catheterization time (after RALP)	≤ 7 days	102 (93.6%)	45 (44.1%)	57 (55.9%)	0.06
	≥8 days	7 (6.4%)	0	7 (100%)	

*min, minimum; max, maximum; BMI, Body Mass Index; iPSA, initial prostate specific antigen; ml, milliliter; IPSS, international prostate symptom score; ICIQ, international consultation of continence questionnaire; incon., incontinence; GS, Gleason Score; NS, nerve-sparing; MMSE, Mini-Mental-State-Examination*.

In the univariate analysis there was no significant difference regarding age at time of surgery (*p* = 0.11), BMI (*p* = 0.55), the prostate volume (*p* = 0.66), and the preoperative quality of micturition and incontinence (IPSS/ICIQ) [*p* = 0.11 (IPSS), *p* = 0.77 (ICIQ)] between the “dry” and “wet” patient-group.

The histopathological characteristics (pT, pN, R status) in the prostatectomy specimen of both groups [wet (≥2 ml) vs. dry (<2 ml) patient group] are comparable. Most patients suffer from a Gleason score 7 (7a/7b), but a locally advanced prostate carcinoma (≥ pT3a). A more advanced carcinoma does not have an influence on postoperative incontinence, since there is no significant difference between the two groups in Gleason score (*p* = 0.5), pT status (*p* = 0.8), nodal status (*p* = 0.57), and resection status (*p* = 0.46). Interestingly, the initial prostate specific antigen (iPSA) value shows a significant difference between “dry” and “wet” patients (*p* = 0.05), with more patients classified wet, when they suffered a higher iPSA.

Patients who performed pelvic floor exercises preoperatively performed equally as well in the MMSE as patients who did not have pelvic floor training (28+/-1.2 vs. 28+/-1.4, *p* = 0.76).

Seven patients had a catheterization time of more than 8 days (insufficient first cystogram). All these patients showed a “wet” pad test result. The catheter duration showed no significance regarding PPI (*p* = 0.06).

Regarding the surgeons' patient populations there was no significant difference between the PPI outcome and the MMSE (Tables 2, 3).

**Table 2:** Distribution of patients in “dry” and “wet” between the surgeons.

**Surgeon**	**Total**	**Dry**	**Wet**	***p*-value**
1	21 (19.3%)	7 (33.3%)	14 (66.7%)	0.56
2	29 (26.6%)	11 (37.9%)	18 (62.1%)	
3	59 (54.1%)	27 (45.8%)	32 (54.2%)	

**Table 3:** Difference between surgeon groups and MMSE distribution.

**MMSE**	**Surgeon 1**	**Surgeon 2**	**Surgeon 3**	***p*-value**
Good	18 (19.8%)	25 (27.5%)	48 (52.7%)	0.56
Intermediate	10 (20.0%)	12 (24.0%)	28 (56.0%)	
Bad	0	1 (20.0%)	4 (80.0%)	

Univariate and multivariate logistic regression analyses identified MMSE results and NS as independent predictors of postoperative incontinence.

Upon multivariable analyses, patients with an intermediate MMSE result had a 3.17 times higher risk of PPI when compared to patients with a good MMSE (95% Confidence Interval (CI): 1.22–9.06, *p* = 0.023), after adjustment for NS (Table 4). Likewise, patients with no NS had a 3.93 times higher risk of PPI (95% CI: 1.54–11.09, *p* = 0.006).

**Table 4:** Univariate and multivariate logistic regression regarding PPI.

	**Outcome**	**ORunivariate**	**ORmultivariate**
MMSE	Good (reference)	1	-
	Intermediate	3.51 (1.40–9.73, *p* = 0.010)	3.17 (1.22–9.06, *p* = 0.023)
	Bad	No OR calculated	No OR calculated
NS (binary)	NS (reference)	1	-
	No NS	3.53 (1.51–8.89, *p* = 0.005)	3.93 (1.54–11.09, *p* = 0.006)

Model diagnostics revealed adequate model calibration and an acceptable discrimination (AUC = 0.744).

## Discussion

UI severely influences daily life, as affected patients report a dramatic loss of their QoL (15). These effects can even lead to mental distress. Avery et al. describe the development of depression in critical cases where UI detrimentally impairs the QoL (16). Since PPI is a well-known and feared side effect of RALP, much effort has been made in the past to understand the physiology of this undesirable situation. The aim was to identify predictors in order to counteract PPI.

Micturition is a voluntary human function based on a complex interaction of the peripheral and central nervous system to coordinate the detrusor muscle and the smooth and striated musculature of the urethra (external sphincter) (17, 18). Since male incontinence has not been researched as extensively as female incontinence, the detailed pathophysiology of PPI remains unclear (2). Nonetheless, surgical and patient risk factors leading to PPI have been identified. Damage to the external sphincter as a surgical cause for PPI seems obvious. Besides the muscle itself, the neurovascular supply can also be impaired by the surgery.

Individual patient-sited parameters identified thus far with significant influence on PPI are older age (19, 20), a shorter (preoperative) membranous urethral length (21), or a bigger gland volume (19, 22), since these factors are associated with less striated muscle tissue (23).

It is proposed that patients with non-modifiable risk factors should be offered more intense targeted preoperative physiotherapy interventions (24). Those interventions focus on training the striated urethral sphincter.

To what extent pelvic floor exercises (pre- and post-operatively) have an influence on the convalescence of continence after radical prostatectomy remains uncertain. Furthermore, the instructions for suitable pelvic floor exercises are not standardized. The traditional “standard of care” includes non-standardized verbal and written instructions to teach standardized pelvic floor exercises (“Kegel-exercises”) (25) before surgery and instructions for the patient to use them after the procedure (26). The AUA 2019 guidelines describe the use of preoperative pelvic floor exercises as follows: “effectiveness of pelvic floor muscle exercise (PFME)/ pelvic floor muscle training (PFMT) has not been definitively shown in the preoperative period.” Nevertheless, the practice continues to be recommended (27).

Overall, sufficient pelvic floor contraction seems to have a substantial impact on postoperative continence. However, training of pelvic floor contraction requires a high level of willingness, coordination, etc. of the patient, as these muscle contractions are “new” to the male patient and such maneuvers are not routinely performed. To our knowledge, no research has been done so far to evaluate how the cognitive status influences the interaction with the muscle-contraction after RALP.

Screening tools for patients' general mental abilities (or disabilities) have so far been insufficient to confirm mental diagnoses. There are various screening tests available, and it is up to the medical professionals to choose the tool they consider most appropriate (28).

However, the MMSE has itself established to be the appropriate tool in clinical practice, as it can also be carried out by physicians who are not neurologically/ psychiatrically experienced. It takes <10 min and has a specificity of 89% with a sensitivity of 81% (28). It has also been shown that the MMSE has a better sensitivity in detecting mild cognitive impairment than other tests, which have a slightly higher overall sensitivity, but are more related to dementia (29).

As described above, the ability of voluntary micturition is a very complex interaction between nervous circles. Voiding happens subconsciously. After radical prostatectomy, the structure of the pelvic floor and the continence mechanisms in men change dramatically. To be able to achieve continence, the patient has to be able to interact with the pelvic floor consciously.

The posed question therefore considers whether sufficient cognitive ability is a prerequisite for effect pelvic floor training to prevent incontinence and/ or to what extent a cognitive deficit could correlate with a PPI.

In our study, the multivariate logistic regression analysis identified the result of the MMSE as an independent predictor of PPI with patients with a worse MMSE having higher risk of UI. Interestingly, the effect size was almost as high as for NS surgery (OR 3.17 vs. 3.93).

The MMSE provides information about the cognitive ability. Therefore, the results suggest that a cognitive deficit is associated with a worse postoperative outcome in terms of continence. It remains unclear whether the increased incontinence rates as a result of cognitive impairment are due to poor execution of the pelvic floor exercises or whether they are associated with reduced body awareness hindering continence training. Further investigations must be performed to distinguish the underlying pathophysiology.

It remains unclear whether the conclusion that pelvic floor training improves post-operative continence can be drawn at all, despite many studies existing up to date. A systematic review from 2020 showed in the long-term analysis that there was no difference in terms of PPI between patients who started pelvic floor exercises immediately postoperatively and the control group that did not perform any exercises (30). Similar results are reported by the 2015 Cochrane Review, in which there was no significant difference in the 12-month continence rate in 2.736 men between those who received PFME and/ or PMFT and those who did not (57% UI in the intervention group and 62% in the control group after 12 months (OR 0.85, 95% CI 0.60–1.22) (31).

A lower cognitive ability should be treated as a non-modifiable parameter just as the anatomical features mentioned above. The fact that an intermediate MMSE result is associated with a worse QoL after RALP, due to PPI, reveals new opportunities in the way patients should be advised. MMSE could be seen as a predictor of the PPI outcome and should lead to a better preparation of the patient before surgery. In our study population, preoperatively performed PFME/ PFMT does not significantly affect the PPI outcome. But this contradicts other studies (2). It could be assumed that patients with a better MMSE performed PFME/ PFMT more frequently before surgery. However, our data shows that there was no difference in MMSE results between patients who carried out preoperative PFME/ PFMT and those who did not.

No studies are available thus far that evaluate cognitive ability and conscious use of the external sphincter urethrae. It would be interesting to evaluate whether poor MMSE results lead to insufficient PFME/ PFMT with subsequent PPI. Implementing the MMSE in the urological clinical “every-day” practice should therefore be the next step.

Our data only refer to the immediate early incontinence after radical prostatectomy. The extent to which early continence is indicative of long-term continence is a controversial dispute. Therefore, it must be shown how the proven correlation reflects in a long-term follow-up.

In this trial there is no difference in incontinence incidence between the high-risk group and the rest of the patient group. We cannot confirm the results of other studies stating that a high-risk prostate cancer constellation is a risk factor for PPI (32), despite the R status in our population.

The natural course of prostate carcinoma with or without therapy forbids establishing the MMSE as a contributing parameter in the decision-making for or against an oncological resection. The RALP is the only therapy that provides a better oncological outcome of the localized disease than watchful waiting (33). Therefore, primarily treating these patients with a different method other than RALP, e.g., by external beam radiation, is not a feasible option, but it can be a tool to help indecisive patients in their treatment choice. In the future, the MMSE may contribute to tipping the scale toward a better postoperative QoL for patients while maintaining a good oncological prognosis, finding its place as a screening tool to identify patients who need more attention in the preoperative phase.

Although this is a prospective study and the first evaluating this research question, there are some limitations worth mentioning: the results only represent the very early continence situation after RALP. Of course, this is the phase of the convalescence where the striated muscle is targeted consciously before transferring it into the unconscious. Further on, the results of this study should be correlated with the long-term incontinence rates. Finally, the patient cohort must be enlarged, for example in a multi-center trial, to avoid potentially underpowered statistical testing and to achieve better reliability of the results.

## Conclusion

The results of this prospective trial suggest that cognitive decline is associated with a worse post-operative outcome in terms of early continence. To our knowledge this study targets this issue for the first time.

A cognitive disability (confirmed by MMSE testing) should be treated as a non-modifiable parameter for PPI. MMSE could be seen as a predictor of the PPI outcome and should lead to a better preparation of the patient before surgery. The results of this study should be compared further on with the long-term incontinence rates.

## Data Availability Statement

The data that support the findings of this study are available from the corresponding author upon reasonable request.

## Ethics Statement

The studies involving human participants were reviewed and approved by the Institutional Review Board of the University Medical Center Goettingen. The patients/participants provided their written informed consent to participate in this study.

## Author Contributions

MR and MM: contributed to conception, design of the study, and wrote the first draft of the manuscript. MM: organized the database. LT, MR, and IP: performed surgery. AU: performed the statistical analysis. All authors contributed to manuscript revision, read, and approved the submitted version.

## Conflict of Interest

The authors declare that the research was conducted in the absence of any commercial or financial relationships that could be construed as a potential conflict of interest.

## Publisher's Note

All claims expressed in this article are solely those of the authors and do not necessarily represent those of their affiliated organizations, or those of the publisher, the editors and the reviewers. Any product that may be evaluated in this article, or claim that may be made by its manufacturer, is not guaranteed or endorsed by the publisher.

## Appendix 1

PAD TEST (13): The pad test was performed as follows:

- Documentation of the pad weight.
- Emptying the bladder prior to test begin.
- Placement of the pad in front of the meatus.
- Asking the patient to:
  ◦ Drink 500 ml water or tea in 20 min,
  ◦ Go for a walk including taking the stairs for 30 min,
  ◦ Cough hard 10 times,
  ◦ Step on the spot for 10 min,
  ◦ Do ten deep squats,
  ◦ Wash their hands with warm water for 1 min, and
  ◦ Micturate and measure the urine volume.
- Ultrasound analysis to evaluate the residual urine volume.
- Weighing of the used pad.

After the pad test, following parameters were documented: Pad weight at the beginning of the test, pad weight at the end of the test, urinary volume micturated, and residual urine volume after emptying the bladder. The loss of urine into the pad was distinguished binarily into the categories “good continence” (<2 ml) and “bad continence” (≥2 ml) (“dry” vs. “wet”). These cut-offs were chosen to distinguish the completely “dry” patients from the rest. Because of the risk of measurement errors, 2 ml were chosen instead of 0–1 ml pad weight difference between the beginning and the end of the test. The same scale was consistently used to measure the pad weight.
